# Evaluation of Lung Volume Reduction in Patients with Interstitial Lung Disease Using Brainomix e-Lung

**DOI:** 10.3390/jcm15062229

**Published:** 2026-03-15

**Authors:** Anton Sabashnikov, Sanjay Agrawal, Bartlomiej Zych, Ihor Krasivskyi, Maria Monteagudo-Vela, Mohamed Osman, Louit Thakuria, Vasiliki Gerovasili, Anand Devaraj, Peter M. George, Anna Reed

**Affiliations:** 1 Guy’s & St Thomas’ NHS Foundation Trust, Royal Brompton and Harefield Hospitals, London SW3 6NP, UK; 2 Department of Cardiac Surgery, University Hospital Bonn, 53127 Bonn, Germany; 3 National Heart and Lung Institute, Imperial College London, London SW7 2AZ, UK

**Keywords:** e-Lung (Brainomix), interstitial lung disease (ILD), lung transplantation, quantitative CT

## Abstract

**Background:** e-Lung (Brainomix) is an artificial intelligence (AI)-driven software that is based on multi-class convolutional neural network (CNN) techniques. The aim of this research was to demonstrate the feasibility of e-Lung to evaluate progression in lung volume reduction in patients with interstitial lung disease (ILD) undergoing lung transplant assessments. **Methods:** This was a single-center retrospective cohort study of consecutive patients with ILD who received lung transplants between June 2021 and November 2024. Patients who underwent serial prospective conventional evaluations using lung function testing (LFT) and conventional radiological assessments as well as retrospective lung volume measurements using e-Lung were included in this study. **Results:** An analysis of 20 consecutive patients who met strict inclusion criteria and underwent an additional e-Lung assessment revealed that both the serial physiological actual total lung capacity (aTLC) measurements and e-Lung-derived lung volume measurements were able to provide recipient lung size estimations and detect serial declines in lung volume. A poorer DLCO (2.61 ± 0.77 vs. 3.87 ± 1.59 mmol/min/kPa, *p* = 0.044) at the time of wait-listing was associated with a significant lung volume reduction. **Conclusions:** e-Lung may serve as an additional upscale tool for the rapid and objective quantitative evaluation of the actual lung volume and the detection of the extent of parenchymal shrinking in patients with advanced ILD awaiting lung transplantation.

## 1. Introduction

Size matching between donors and recipients is a major challenge in lung transplantation, particularly in patients with progressive restrictive lung diseases, such as interstitial lung disease (ILD). Current methods to assess the recipient lung volume include physiological assessments of the total lung capacity (TLC) and clinician assessments of imaging modalities. Physiological testing demonstrates intra-individual variability in end-stage fibrotic lung disease, and imaging interpretation is subjective and prone to inter-observer variability. The inaccurate prediction of recipient lung volumes significantly increases operative transplant mortality, and, in some cases, the physiological assessment underestimates the TLC and may preclude suitable patients from being waitlisted altogether. There is an urgent need for robust, objective measurements of the recipient lung volume in the context of lung transplants.

e-Lung (Brainomix) is an artificial intelligence (AI)-driven software based on multi-class convolutional neural network (CNN) techniques that can be applied to standard CT scans. e-Lung has demonstrated the ability to predict the progression of ILD [1,2]; however, to date it has not been evaluated for the assessment of the TLC in the context of lung transplantation. e-Lung provides automated measurements of the total and peripheral lung volume in patients with ILD based on CT scans. This feature has important potential as an adjunct to objective decision-making in terms of size matching at the time of lung transplantation [3,4]. The current practice is to combine visual radiological and physiologic assessments for patients with ILD to evaluate recipients’ lung volumes [3,5]. However, both approaches may be associated with limitations related to either physiological or assessment biases. In fact, lung function testing (LFT), by virtue of it being volitional, may be not reliable due to patient underperformance, and the radiological visualization may also be subjective. Therefore, further studies deriving patients’ lung volumes from CT scans for a more accurate evaluation of lung size matching are required.

The aim of this pilot retrospective cohort study was to demonstrate the feasibility of e-Lung as an adjunct to conventional methods for the assessment of recipient lung volumes for improved donor–recipient matching.

## 2. Methods

This was a retrospective single-center cohort study conducted at a tertiary lung transplant center. The Institutional Review Board determined that formal ethical approval was not required under English law for purely retrospective clinical analyses. Patient consent was waived due to the retrospective nature of this study.

**Patient Selection**

Adult patients with end-stage ILD who underwent lung transplantation between June 2021 and November 2024 were screened.

**Inclusion criteria:**

- Confirmed diagnosis of ILD;
- At least two serial LFT assessments, including FVC, DLCO, and aTLC;
- Corresponding thoracic CT scans performed within ±6 weeks of each LFT session;
- Minimum interval of approximately one year between serial assessments.

**Exclusion criteria:**

- Missing complete LFT data;
- Absence of appropriately timed CT imaging;
- Incomplete imaging quality preventing automated analysis.

Only patients who successfully underwent transplantation during the study period were included.

After applying criteria, 20 consecutive patients were included.

**Imaging Analysis**

All CT thorax scans were processed using e-Lung software (Brainomix), which performs automated lung segmentation and quantitative volume estimation. Total lung volume was extracted for analysis. Imaging analysis was performed retrospectively and blinded to physiological results.

**Physiological Assessment**

LFT was performed according to international standards. aTLC was considered the conventional reference measure. Predicted TLC (pTLC) was calculated using established sex-specific formulae.

**Study Objectives**

***Primary objective***

The primary objective of this project was to evaluate and quantify lung volumes estimated by e-Lung and compare these to conventional aTLC measurements during physiological LFT as a current gold standard.

*Statistics*

All data was analyzed using Statistical Package for Social Sciences, version 25.0 (SPSS Inc., Chicago, IL, USA), and presented as continuous or categorical variables. The continuous data were evaluated for normality of distribution using histograms and confirmed with one-sample Kolmogorov–Smirnov test. Univariate analysis was performed using either Student’s *t*-test or Mann–Whitney U test for normally distributed and skewed continuous variables, respectively. Pearson’s χ^2^ or Fisher exact tests were applied for categorical data dependent on the minimum expected count in each cross-tab. Data were expressed as the mean ± standard deviation in cases of normally distributed or median (interquartile range) skewed continuous variables. The categorical data were presented as total numbers and percentages. A paired *t*-test, Wilcoxon signed rank test, or Mann–Whitney test were used for statistical comparison of continuous data over time for normally distributed and skewed data, as appropriate. *p* < 0.05 was considered statistically significant, whereas 0.05 ≤ *p* < 0.1 was defined as statistical trend.

## 3. Results

The mean age of the patient cohort was 47.2 ± 12.8 years at the first LFT and CT thorax evaluation, 49.2 ± 12.6 years at the subsequent LFT and CT thorax evaluation, and 49.9 ± 12.4 years at the time of censoring. The mean height of the patients was 172.2 ± 10.7 cm; the mean predicted total lung capacity (pTLC) was 6389 ± 1397 mL, and 35% (n = 7) of patients were female. The calculation of the pTLC was performed using the equation (9.4 × height in m) − (0.015 × age in years) − 9.167 for male patients and (7.9 × height in m) − (0.008 × age in years) − 7.49 for female patients [6]. As all patients on the waiting list had to be non-smokers; 65% (n = 13) of patients had never smoked, whereas 35% (n = 7) of patients had a history of smoking.

Comparing actual lung volume measurements over serial evaluations using both the conventional LFT (aTLC) and e-Lung software (automated lung volume measurements) applied to high-resolution CT thorax scans showed no statistically significant differences in lung volumes at either the first (*p* = 0.554) or subsequent (*p* = 0.914) evaluation. The mean difference in the lung volume between the two methods revealed a 69 ± 515 mL difference at the first evaluation and a 18 ± 706 mL difference at the subsequent evaluation, with a relative overestimation tendency evident in the LFT.

The serial LFT revealed a mean aTLC of 3913 ± 878 mL and 3647 ± 1023 mL at the first and subsequent evaluation, with a median time difference between these two measurements of 478 (210; 904) days. The mean decrease in the aTLC was 266 ± 752 mL (*p* = 0.140). There was a statistically significant difference between the pTLC and aTLC at both the first and subsequent LFT evaluation (*p* < 0.001), as expected due to the nature of the disease.

The serial lung volume evaluations using the e-Lung software revealed a total lung volume of 3879 ± 987 mL and 3618 ± 1042 mL at the first and subsequent evaluation of the high-resolution CT thorax scans performed 431 (293; 897) days apart. The mean automated lung volume drop accounted for 262 ± 542 mL (*p* = 0.043).

All parameters obtained during baseline and follow-up evaluations using both the LFT and e-Lung software, as well as quantitative changes over time and their significance, are presented in Table 1.

A subgroup analysis of patients with a >10% lung volume reduction based on the aTLC between two serial e-Lung evaluations, which occurred in half of the entire cohort, was performed to assess risk factors for rapid volume reductions. When analyzing the potential impact of initial conventional LFT results, no significance could be observed regarding the FVC (*p* = 0.891); however, a poorer D_LCO_ (2.61 ± 0.77 vs. 3.87 ± 1.59 mmol/min/kPa, *p* = 0.044) appeared to be significantly associated with a lung volume reduction >10% over time. Neither gender (*p* = 0.888) nor a history of smoking (*p* = 0.279) were associated with a >10% lung volume loss (Table 2).

As expected, in patients with advanced ILD, donor lungs were oversized relatively to the recipients’ aTLC during transplantation, with the mean donor pTLC accounting for 5683 ± 11,402 mL, which was significantly higher than both recipients’ aTLC (3647 ± 1023 mL) and lung volume measured using e-Lung (3618 ± 1042 mL) at the latest timepoint before transplantation (*p* = 0.001 and *p* = 0.004, respectively).

## 4. Discussion

Lung transplantation has remained the gold standard treatment for end-stage lung failure over decades [7,8]. Despite improvements in outcomes, transplant teams have faced significant challenges associated with donor organ shortages, morbidity and mortality on the waiting list, and appropriate size matching, particularly in ILD patients.

The accurate assessment of the recipient lung volume in these patients undergoing lung transplantation is both challenging and crucial. Profound structural changes, such as progressive fibrosis and heterogeneous remodeling, distort the pulmonary anatomy and make volumetric measurements less reliable using conventional imaging or functional physiological tests. Nevertheless, a precise lung volume evaluation is vital for optimal donor–recipient size matching and the prevention of post-transplant complications, given that mismatches can influence graft function, long-term outcomes, and survival after a transplantation in ILD.

We hypothesized that e-Lung could be an additional reliable tool for the accurate assessment of lung volume as an adjunct to traditional methods. Whereas automated lung volume measurements in idiopathic pulmonary fibrosis have been reported on in the previous literature, there is a lack of evidence directly related to lung transplantation [9,10].

Due to the disease-related progressive reduction in the lung volume and pleural space in patients with advanced ILD requiring lung transplantation, size matching represents one of the most complex clinical and logistical issues [11]. The frequently observed overestimation of the real pleural space size in ILD patients may lead to poorer outcomes or the need for additional surgical interventions, such as lung volume reduction surgery or delayed chest closure [12,13]. On one hand, dynamic changes in the lung volume require close monitoring; on the other hand, all conventional methods of pleural space size estimation remain semi-reliable and frequently inaccurate [5,14]. To date, there has been no evidence regarding the predictability of parenchymal changes leading to lung volume reductions over time. While there has been debatable evidence in the literature and conflicting expert opinions regarding size matching in ILD, the most frequently used quantitative matching approach has been based on the formula summarized by Barnard et al. [3]. This approach requires the accurate estimation of the actual current lung volume, usually in the form of the aTLC obtained during LFT. However, conventional radiological and functional estimations have been shown to be associated with potential serious limitations, making the assessment accuracy very limited [3,15]. Indeed, LFT results may not be sufficiently conclusive due to the difficulty some patients with advanced lung disease experience in performing the test reproducibly, whereas pleural space measurements on the CT scan may be affected by interpretation bias. To increase the reliability of this estimation, both diagnostic modalities have been utilized, despite their limitations, for more optimal matching.

In this study, automated CT lung volume assessment technology was analyzed regarding its potential clinical usefulness to accurately estimate the actual size of the diseased lungs and to detect changes in lung volume over time, compared to conventional methods. A potential benefit of the e-Lung-based lung volume estimation may be that the use of quantitative CT tools reduces the risk of human inter-observer variability [16]. Also, physiological confounders frequently observed due to poor patient performance during LFT, affecting the reliability of aTLC measurements, could be potentially diminished using e-Lung. However, patient performance can also affect the inspiratory effort when conducting CT.

Based on the results of this study, e-Lung may be an additional source for size matching, providing similarly accurate lung volume sizes to LFT. Also, it may be a sensitive tool for detecting changes in size over time in addition to conventional aTLC measurements, similarly demonstrating the extent of the lung volume reduction over time.

e-Lung may have clinical utility as an adjunctive strategy for conventional methods, particularly taking into consideration the high variability of ILD specifications and the associated degree and progression of lung shrinking and in frequently occurring combined pathologies (i.e., restrictive and obstructive components) leading to partial hyperinflation. Such complex pathologies may be an additional significant challenge in terms of lung volume estimation, as shown in previous research [16]. While at this stage e-Lung cannot replace conventional LFT for size matching in lung transplantation, it has the potential to be used as a clinical monitoring tool for estimating actual lung volumes in recipients.

In order to provide more robust and reproducible matching, e-Lung has the potential to correct conventional methods of lung volume estimation and enable transplant teams to more accurately match donor organs, potentially improving outcomes. Conversely, a poorer D_LCO_ may be a warning sign of a potentially rapid lung volume size decline over time, requiring more close monitoring. Those patients, particularly of small body habitus, may have to be prioritized and transplanted sooner rather than later to avoid reaching the point of untransplantability due to the inability to match donor lungs if the recipient’s lung volume decreases to a level that is too low for a real chance to find an appropriate donor match. Even more important is the estimation of lung size decline in small patients with O type blood, as their waiting times are usually longer. In this same way, shorter waiting times may help combat the battle against the clock that is linked to the permanent lung size decline associated with ILD progression.

## 5. Conclusions

e-Lung volume measurement is associated with a similar lung volume estimation compared to conventional aTLC evaluations in LFT. Also, this software may be an additional tool for evaluating the extent of the reduction in lung volume over time in ILD patients on the waiting list. At the same time, the D_LCO_ measured during conventional LFT may have significant predictive value for significant lung volume reductions over time in patients with ILD.

## 6. Limitations of This Study

This was a single-center evaluation. The sample size was small due to strict inclusion criteria and the need for serial and timely arranged radiological and functional evaluations. For that reason, no statistical power calculation was performed, as all patients meeting inclusion criteria and fulfilling e-Lung software requirements were included in this study. This initial experience should encourage further larger multi-center studies.

## 7. Future Research Directions

Future prospective multi-center studies are required to:

- Validate the agreement between AI-derived lung volumes and intraoperative findings;
- Correlate volumetric changes with perioperative and post-transplant outcomes;
- Evaluate reproducibility across CT acquisition protocols;
- Assess the integration of automated volumetry into donor–recipient allocation algorithms.

Larger cohorts would also allow for the development of predictive models incorporating the DLCO and CT-derived metrics to identify patients at risk of rapid pleural space reduction.

## Figures and Tables

**Table 1:** Recipients’ baseline and follow-up characteristics.

	Baseline	Follow-Up	*p* Value
FVC (mL)	2390 ± 581	2157 ± 548	0.024
aTLC (mL)	3913 ± 878	3647 ± 1023	0.140
D_LCO_ (mmol/min/kPa)	3.01 ± 1.28	2.51 ± 1.20	0.060
e-Lung volume (mL)	3879 ± 987	3618 ± 1042	0.043

*p* < 0.05 was considered statistically significant. FVC, forced vital capacity; aTLC, actual total lung capacity; and D_LCO_, diffusing capacity of the lungs for carbon monoxide.

**Table 2:** Impact of baseline parameters on lung volume reduction over time.

	≤10%	>10%	*p* Value
Female (%)	36.4	33.3	0.888
Ex-smoker (%)	45.5	22.2	0.279
FVC (mL)	2373 ± 604	2410 ± 587	0.891
DLCO (mmol/min/kPa)	3.87 ± 1.59	2.61 ± 0.77	0.044

*p* < 0.05 was considered statistically significant. FVC, forced vital capacity; DLCO, diffusing capacity of the lungs for carbon monoxide.

## Data Availability

Data can be obtained via a special request.

## Abbreviations

The following abbreviations are used in this manuscript:

AI	artificial intelligence
CNN	convolutional neural network
ILD	interstitial lung disease
LFT	lung function testing
aTLC	actual total lung capacity
TLC	total lung capacity
FVC	forced vital capacity
D_LCO_	carbon monoxide
